# Anti-malarial prescription practices among outpatients with laboratory-confirmed malaria in the setting of a health facility-based sentinel site surveillance system in Uganda

**DOI:** 10.1186/1475-2875-12-252

**Published:** 2013-07-19

**Authors:** David Sears, Ruth Kigozi, Arthur Mpimbaza, Stella Kakeeto, Asadu Sserwanga, Sarah G Staedke, Michelle Chang, Bryan K Kapella, Denis Rubahika, Moses R Kamya, Grant Dorsey

**Affiliations:** 1 Department of Medicine, San Francisco General Hospital, University of California, 1001 Potrero Avenue, SFGH Building 30, Room 3300, Box 0811, San Francisco, CA 94143, USA; 2 Uganda Malaria Surveillance Project, Kampala, Uganda; 3 Child Health & Development Centre, Makerere University College of Health Sciences, Kampala, Uganda; 4 London School of Hygiene and Tropical Medicine, London, UK; 5 Malaria Branch, Centers for Disease Control and Prevention, Atlanta, GA, USA; 6 Uganda Ministry of Health, Kampala, Uganda; 7 Department of Medicine, Makerere University College of Health Sciences, Kampala, Uganda

**Keywords:** Malaria, Treatment, Outpatient, Anti-malarial, Artemisinin-based combination therapy, *Plasmodium falciparum*, Artemether-lumefantrine, Prescription, Case management

## Abstract

**Background:**

Most African countries have adopted artemisinin-based combination therapy (ACT) as the first-line treatment for uncomplicated malaria. The World Health Organization now recommends limiting anti-malarial treatment to those with a positive malaria test result. Limited data exist on how these policies have affected ACT prescription practices.

**Methods:**

Data were collected from all outpatients presenting to six public health facilities in Uganda as part of a sentinel site malaria surveillance programme. Training in case management, encouragement of laboratory-based diagnosis of malaria, and regular feedback were provided. Data for this report include patients with laboratory confirmed malaria who were prescribed anti-malarial therapy over a two-year period. Patient visits were analysed in two groups: those considered ACT candidates (defined as uncomplicated malaria with no referral for admission in patients ≥ 4 months of age and ≥ 5 kg in weight) and those who may not have been ACT candidates. Associations between variables of interest and failure to prescribe ACT to patients who were ACT candidates were estimated using multivariable logistic regression.

**Results:**

A total of 51,355 patient visits were included in the analysis and 46,265 (90.1%) were classified as ACT candidates. In the ACT candidate group, 94.5% were correctly prescribed ACT. Artemether-lumefantrine made up 97.3% of ACT prescribed. There were significant differences across the sites in the proportion of patients for whom there was a failure to prescribe ACT, ranging from 3.0-9.3%. Young children and woman of childbearing age had higher odds of failure to receive an ACT prescription. Among patients who may not have been ACT candidates, the proportion prescribed quinine *versus* ACT differed based on if the patient had severe malaria or was referred for admission (93.4% *vs* 6.5%) or was below age or weight cutoffs for ACT (41.4% *vs* 57.2%).

**Conclusions:**

High rates of compliance with recommended ACT use can be achieved in resource-limited settings. The unique health facility-based malaria surveillance system operating at these clinical sites may provide a framework for improving appropriate ACT use at other sites in sub-Saharan Africa.

## Background

In 2001 the World Health Organization (WHO) recommended artemisinin-based combination therapy (ACT) for the treatment of uncomplicated *Plasmodium falciparum* malaria in countries with resistance to older monotherapies [1]. At the time ACT was neither widely available nor affordable in many countries with limited resources. Five years later, 41 African countries (and 65 worldwide) had adopted ACT as first-line therapy and 21 were deploying ACT at public health facilities [2]. In 2010, WHO expanded its recommendations to include laboratory confirmation with microscopy or a rapid diagnostic test (RDT) before initiating anti-malarial therapy [3]. The use of ACT based on laboratory confirmation represents a dramatic shift in malaria case management in Africa following decades of largely empiric therapy with inexpensive, older monotherapies. Currently, limited data exist on recent anti-malarial prescription practices in Africa following these changes. In Kenya, where case management practices have been published following a policy change promoting universal parasitological diagnosis before treatment, a majority of patients were not getting diagnostic testing or treatment according to test result, although those with positive tests and uncomplicated malaria did receive recommended treatment with artemether-lumefantrine (AL) in 90% of cases when AL was available [4].

Uganda, which has some of the highest rates of malaria transmission in the world [5,6], adopted ACT in 2004 and began deployment in early 2006 [7]. AL was selected as the first-line therapy for uncomplicated malaria and artesunate plus amodiaquine was recommended as an alternative first-line therapy. Oral quinine was recommended as second-line therapy for uncomplicated malaria and parenteral quinine remained first-line therapy for complicated malaria [8,9]. In 2006, training was conducted throughout the country with the goal of educating all front-line clinicians on the new treatment policy [10]. Job aids and clinical guidelines were also distributed at the health facilities. In Uganda, clinical consultation, laboratory testing, and medications are provided free of charge at all public health facilities.

In 2006, with support from the U.S. President’s Malaria Initiative (PMI), a unique sentinel site malaria surveillance programme was established at six public health facilities in Uganda. As described previously, this programme was successful in reaching a 97% laboratory testing rate for patients with suspected malaria and fewer than 10% of patients with a negative laboratory test were prescribed anti-malarial therapy [11]. The purpose of this report is to describe anti-malarial prescription practices among outpatients with laboratory-confirmed malaria and to identify factors associated with inappropriate therapy. A better understanding of how Uganda’s change in malaria treatment policy has translated into practice in the setting of an active training and surveillance programme is vital to improving the appropriate use of anti-malarial therapy.

## Methods

### Health facility-based malaria surveillance system

**Figure 1:**
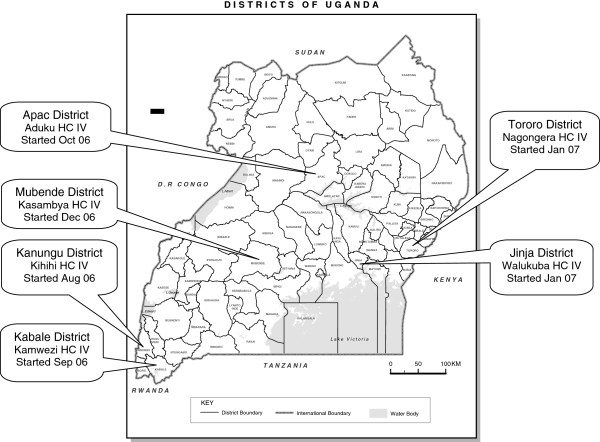
Location of sentinel site malaria surveillance programme health facilities.

The Uganda Malaria Surveillance Project (UMSP) is a collaboration between Makerere University, the Uganda Ministry of Health, the London School of Hygiene and Tropical Medicine, and the University of California San Francisco. In 2006 UMSP, working with the Uganda National Malaria Control Programme (NMCP), created a health facility-based malaria surveillance system at six sentinel sites throughout the country, the details of which have been described previously [11,12]. Briefly, these facilities are level IV government-run health centers which primarily provide outpatient services, have catchment populations of approximately 100,000 people, and were selected to represent a range of settings with respect to malaria epidemiology (Figure 1). Most patients are treated by clinical officers, but medical officers, nurses, and midwives may also evaluate patients. Each site has a pharmacy and a laboratory with capacity to perform microscopy. There are also approximately 10–15 inpatient beds, which may be utilized for brief admissions, although this capacity is not available at two of the sites (Kasambya and Walukuba).

Individual-level data are collected for each outpatient who presents to the health facility using a standardized case record form (CRF) completed by the clinicians during the patient encounter (Additional file 1). Data collected include demographics, malaria laboratory test results (microscopy or RDT), diagnoses (including uncomplicated malaria, severe malaria, or malaria in pregnancy), medications prescribed and dispensed, and disposition. Suspected malaria is defined as all patients referred for malaria laboratory testing plus all patients not referred for a malaria laboratory test but given a clinical diagnosis of malaria. The dosing of each medication and the route of administration is not recorded.

### Supply of anti-malarial medications

Most medications are purchased by the Ministry of Health with government funds and financial support from the Global Fund for AIDS, Tuberculosis and Malaria (GFATM). International donors have also made relatively smaller quantities of anti-malarials available. At the start of the study the Uganda National Medical Stores distributed medications to each district every two months based on the amount of medication requested by the district [9]. The district would then distribute the medications to the health facilities. In mid-2011, due to frequent stock-outs, the National Medical Stores began distributing medications directly to the health facilities. For level IV health facilities the amount of medication delivered was still dependent on the amount requested. The Uganda Ministry of Health has also worked with non-governmental partners to improve forecasting of stock-outs and to streamline the anti-malarial supply chain. These efforts have led to substantial reductions in anti-malarial stock-outs [13]. Notably, the supply of anti-malarials is not influenced by UMSP staff.

### Sentinel site health care worker training and other support

Following the initiation of the malaria surveillance programme in 2006, 118 clinicians from the six sentinel sites and three additional sites underwent a six-day training designed to promote a high-quality team-based approach to fever case management. Clinical training involved lectures and simulated case-based training centered on the Uganda Ministry of Health malaria treatment guidelines as previously described [14,15]. Following the initial training programme, the sentinel sites were visited by the UMSP staff every 1–2 months to provide feedback on case management, share data, and ensure the supply of laboratory materials. From 2011 to 2012, site visits were spaced out to every 4–6 months.

### Statistical analysis

Data collected over a 2-year period from January 2011 through December 2012 were included in this study. Only patients with laboratory-confirmed malaria, documented prescription of an anti-malarial, and documented age were included in the analysis. Patients recorded as being pregnant were excluded as the surveillance system does not reliably capture the trimester of pregnancy and thus it was not possible to classify these patients based on their candidacy for ACT. For analysis, study participants were placed into two groups based on whether or not it was thought they were candidates for ACT in accordance with national malaria treatment guidelines. Those who were considered candidates for ACT were given a diagnosis of uncomplicated malaria, were four months of age or older, were 5 kg in weight or above, and were not referred for admission. Those who were not clear candidates for ACT were either given a diagnosis of severe malaria, referred for admission, less than four months of age, or had a weight recorded as <5 kg.

Statistical analysis was performed using Stata 12.0 (Stata Corp, College Station, TX). Associations between variables of interest and inappropriate anti-malarial therapy among patients in the ACT candidate group were estimated using logistic regression. Variables of interest included clinical site, age, whether or not the patient was a woman of child-bearing age (15 to 49 years), concurrent prescription of antibiotics, and transmission season (where high transmission season was defined as the months at each clinical site where the malaria test positivity rate is above the mean for that site and low transmission season comprised the remaining months).

## Results

### Study population and characteristics

**Figure 2:**
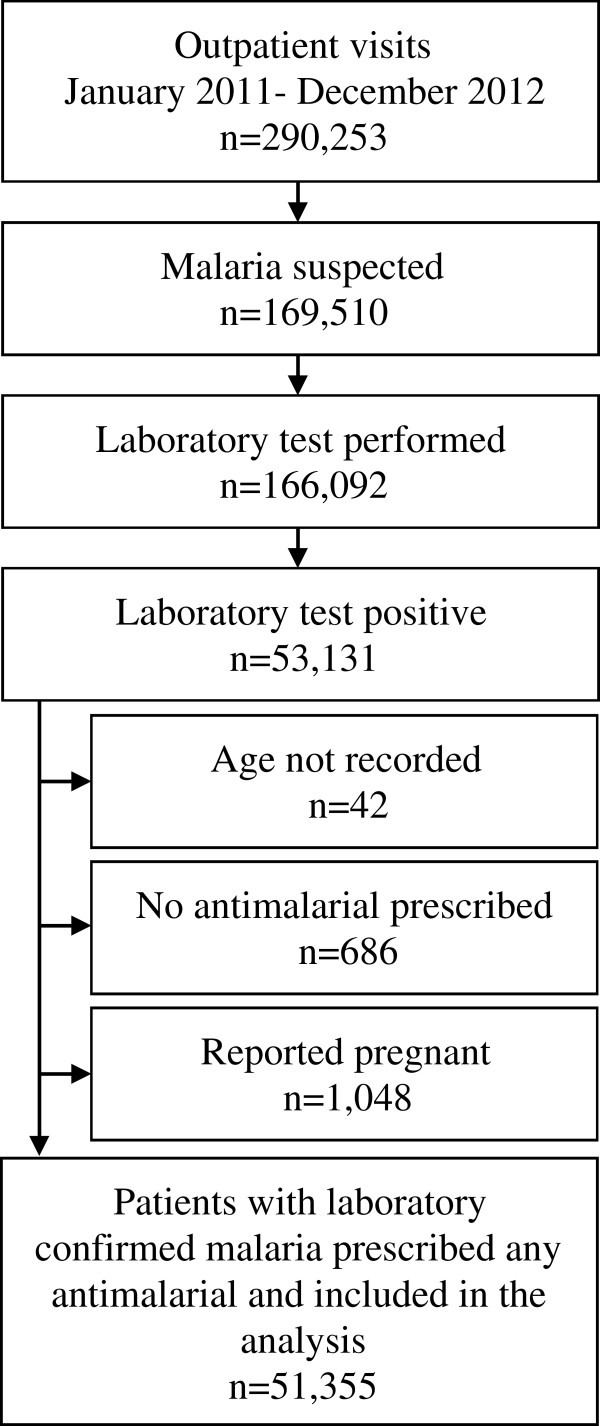
Selection of patient visits included in the analysis.

During the study period a total of 290,253 outpatient visits were recorded (Figure 2). Malaria was suspected in 58.4% of all outpatient visits (range 38.5-74.6% across the sites). Among patients with suspected malaria, 98.0% underwent diagnostic testing. Among those who underwent diagnostic testing, only 3.2% had an RDT performed, with over 93% of RDTs performed at two sites (Kasambya and Kamwezi). Of all patients who underwent diagnostic testing, 32.0% had laboratory-confirmed malaria (range 23.8-38.9% across the sites). Within the group of patients with laboratory-confirmed malaria, 3.3% were excluded from the final analyses: 2.0% were reported to be pregnant, 1.3% had no prescribed anti-malarial drugs recorded, and <0.1% had no age recorded. Of note, among patients who were reported to be pregnant, 54% were prescribed ACT, 45% were prescribed quinine, and 1% were prescribed other anti-malarials.

Characteristics of the 51,355 outpatient visits included in the final study population are described in Table 1. The proportion of patients <5 years of age ranged from 20.8% in Kamwezi to 63.8% in Nagongera. The study population showed a slight female predominance, which was consistent across sites. Overall 19.6% of patients were women of childbearing age (range 11.6-24.0% across the sites). The concurrent prescription of antibiotics with anti-malarials was recorded in 29.1% of patients, ranging from 10.2% in Kamwezi to 55.7% in Aduku.

**Table 1:** Characteristics of the study population

**Variable**	**All Sites**	**Aduku**	**Kamwezi**	**Kasambya**	**Kihihi**	**Nagongera**	**Walukuba**
Patient visits, n (% total)	51,355	4,489 (8.7%)	5,241 (10.2%)	11,227 (21.9%)	9,076 (17.7%)	8,880 (17.3%)	12,442 (24.2%)
Age in years, median (IQR^a^)	8 (2.6-20)	9 (2.4-23)	12 (5.0-23)	10 (3.0-22)	8 (3.5-17)	2.5 (1.2-10)	11 (3.8-24)
Female gender, n (%)	29,025 (56.5%)	2,583 (57.5%)	2,794 (53.3%)	6,426 (57.2%)	5,160 (56.9%)	4,842 (54.5%)	7,220 (58.0%)
Women of childbearing age^b^, n (%)	10,050 (19.6%)	1,078 (24.0%)	1,159 (22.1%)	2,332 (20.8%)	1,594 (17.6%)	1,031 (11.6%)	2,856 (23.0%)
Prescribed antibiotics, n (%)	14,966 (29.1%)	2,500 (55.7%)	534 (10.2%)	5,163 (46.0%)	2,422 (26.7%)	1,744 (19.6%)	2,603 (20.9%)
During high transmission season^c^, n (%)	30,435 (59.3%)	2,629 (58.6%)	3,142 (60.0%)	6,469 (57.6%)	5,123 (56.5%)	5,220 (58.8%)	7,852 (63.1%)

^a^ Inter-quartile range.

^b^ 15-49 years of age.

^c^ High transmission season: any month where the test positivity rate is above the mean test positivity rate for the site.

### Prescription practices among ACT candidates

Considering all patient visits included in the final analyses with laboratory-confirmed malaria who were prescribed an anti-malarial, 90.1% were considered to be candidates for ACT, ranging from 72.8% in Nagongera to 99.5% in Walukuba (Table 2). ACT candidates were appropriately prescribed ACT at 94.5% of visits. Among patients who were prescribed ACT, 97.3% were prescribed the first-line regimen AL (n = 42,537). The remaining patients were prescribed artemisinin-naphthoquine (AN, n = 870, 2.0%), dihydroartemisinin-piperaquine (DP, n = 326, 0.7%), and a single patient was prescribed artesunate plus sulphadoxine-pyrimethamine (SP). Over 90% of AN prescriptions and over 95% of DP prescriptions originated from single sites (Kasambya and Kamwezi respectively). Of note, no patients were prescribed artesunate plus amodiaquine, which is considered an alternative first-line regimen in Uganda. When AL was prescribed, it was recorded as dispensed by the pharmacy at 92.0% of visits in 2011 and 99.0% of visits in 2012. Other forms of ACT were dispensed after prescription at 99.0% of visits.

**Table 2:** Classification of candidacy for ACT by clinical site

**Candidacy for ACT**	**All Sites**	**Aduku**	**Kamwezi**	**Kasambya**	**Kihihi**	**Nagongera**	**Walukuba**
Candidate for ACT, n (%)	46,265 (90.1%)	4,039 (90.0%)	4,981 (95.0%)	11,127 (99.1%)	7,274 (80.2%)	6,464 (72.8%)	12,380 (99.5%)
May not be candidate for ACT, n (%)	5,090 (9.9%)	450 (10.0%)	260 (5.0%)	100 (0.9%)	1,802 (19.9%)	2,416 (27.2%)	62 (0.5%)
Severe malaria or referred for admission	4,716	390	247	8	1,760	2,305	6
Age <4 months or weight <5 kg	374	60	13	92	42	111	56

Among the 2,531 clinic visits where patients were classified as ACT candidates but were not prescribed ACT, anti-malarials prescribed included quinine (n = 2,396, 94.7%), artemether (n = 128, 5.1%), chloroquine plus SP (n = 3, 0.1%), chloroquine (n = 2, <0.1%), artesunate (n = 1, <0.1%) and SP (n = 1, <0.1%). Associations between variables of interest and failure to prescribe ACT are presented in Table 3. The proportions of patients classified as ACT candidates but not prescribed ACT ranged from 3.0% in Aduku to 9.3% in Nagongera. Compared to Aduku, the other sites had significantly higher odds of not prescribing ACT after controlling for other variables, with odds ratios ranging from 1.43 (95% CI 1.16-1.76) in Walukuba to 2.60 (95% CI 2.12-3.20) in Nagongera. Decreasing age was associated with increasing odds of not being prescribed ACT. Patients 4 months to <2 years of age had over four times the odds of not being prescribed ACT (OR = 4.69, 95% CI 4.10-5.37) compared to patients 10 years of age or older after controlling for other variables. Women of childbearing age had higher odds of not being prescribed ACT (OR = 1.79, 95% CI 1.54-2.07). Patients who were prescribed antibiotics had lower odds of not being prescribed ACT (OR = 0.89, 95% CI 0.81-0.98). There was no significant association between malaria transmission season and the failure to prescribe ACT.

**Table 3:** Variables associated with failure to prescribe ACT to patients who are candidates for ACT

**Variable**	**Category**	**Proportion with ACT not prescribed**	**Unadjusted**		**Adjusted**	
			**OR (95% CI)**	**p-value**	**OR (95% CI)**	**p-value**
Clinical site	Aduku	3.0%	1.0 (reference)	-	1.0 (reference)	-
	Walukuba	4.1%	1.41 (1.15-1.73)	0.001	1.43 (1.16-1.76)	0.001
	Kamwezi	4.2%	1.46 (1.16-1.83)	0.001	1.68 (1.34-2.13)	<0.001
	Kihihi	5.6%	1.96 (1.59-2.41)	<0.001	2.11 (1.71-2.61)	<0.001
	Kasambya	6.2%	2.17 (1.78-2.64)	<0.001	2.24 (1.83-2.73)	<0.001
	Nagongera	9.3%	3.36 (2.75-4.11)	<0.001	2.60 (2.12-3.20)	<0.001
Age	≥10 years	3.1%	1.0 (reference)	-	1.0 (reference)	-
	≥5 years and <10 years	5.3%	1.71 (1.51-1.93)	<0.001	2.19 (1.89-2.54)	<0.001
	≥2 years and <5 years	7.9%	2.63 (2.35-2.93)	<0.001	3.33 (2.90-3.82)	<0.001
	≥4 months and <2 years	11.0%	3.80 (3.43-4.23)	<0.001	4.69 (4.10-5.37)	<0.001
Women of childbearing age^a^	No	5.8%	1.0 (reference)	-	1.0 (reference)	-
	Yes	4.2%	0.70 (0.63-0.78)	<0.001	1.79 (1.54-2.07)	<0.001
Prescribed antibiotics	No	5.5%	1.0 (reference)	-	1.0 (reference)	-
	Yes	5.4%	0.98 (0.90-1.07)	0.674	0.89 (0.81-0.98)	0.017
Transmission season	Low	5.3%	1.0 (reference)	-	1.0 (reference)	-
	High	5.6%	1.04 (0.96-1.13)	0.302	1.08 (0.99-1.17)	0.084

^a^Women 15 to 49 years old.

### Prescription practices among patients who may not have been ACT candidates

**Figure 3:**
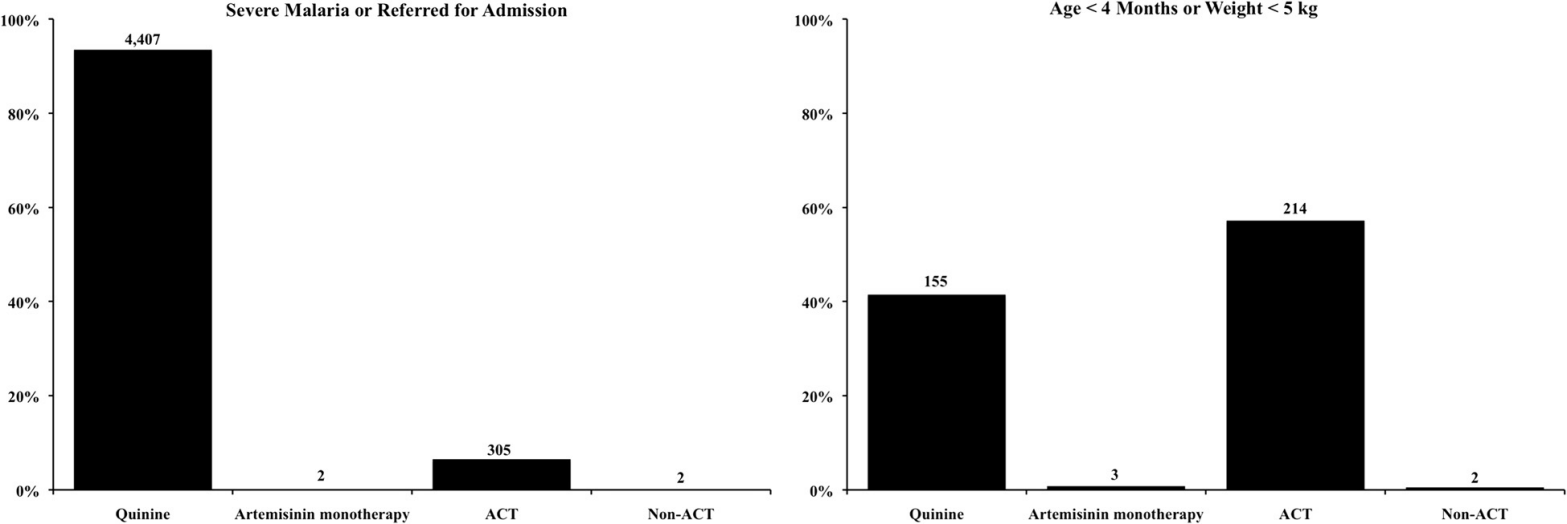
Prescription practices among patients who may not have been ACT candidates.

Of the 5,090 patients who may not have been candidates for ACT, 92.7% were classified as such due to being given a diagnosis of severe malaria or referred for admission (Table 2). The remaining patients were below the recommend age or weight requirements for administration of ACT. Among sites with access to onsite inpatient facilities, the proportion of patients that were given a diagnosis of severe malaria or referred for admission ranged from 4.7% in Kamwezi (where 20.8% of study patients were under five years-old) to 26.0% in Nagongera (where 63.8% of study patients were under five years-old). In contrast, at the two sites that lacked access to onsite inpatient facilities (Kasambya and Walukuba), less than 1% of patients were given a diagnosis of severe malaria or referred for admission. Prescribing practices for the study participants who may not have been candidates for ACT differed based on their reason for inclusion in the non-ACT group (Figure 3). Those given a diagnosis of severe malaria or referred for admission were most frequently prescribed quinine (93.4%), followed by ACT (6.5%). Children below the age of four months or under 5 kg in weight were prescribed quinine less frequently than ACT (41.4% *vs* 57.2%). In both groups the ACT prescribed was nearly always AL (n = 512, 98.7%), while a minority of patients received prescriptions for DP (n = 5, 1.0%) and AN (n = 2, 0.4%). There were five patients across the two groups who were prescribed artemisinin monotherapies (artemether = 4, artesunate = 1) and four who were prescribed non-ACT (chloroquine = 3, SP = 1). When quinine was prescribed it was recorded as dispensed at 90.4% of visits in 2011 and 97.0% of visits in 2012. ACT was dispensed after prescription at 89.5% of visits in 2011 and 97.8% of visits in 2012.

## Discussion

Changes in malaria case management advocated by WHO, including the use of ACT and treatment based on laboratory confirmation, have the potential to efficiently reduce the burden of malaria in Africa where health systems are strained. Results from this study, where nearly all malaria suspects received a diagnostic test and 94.5% of ACT candidates were prescribed ACT for laboratory-confirmed malaria, demonstrate that high rates of appropriate malaria case management can be achieved in outpatient settings. Much of this success is likely due to the unique health facility-based malaria surveillance system, which included an additional training programme for clinicians and periodic feedback sessions where data were shared and progress toward meeting case management goals was tracked. The impressive rates of appropriate ACT use at these sites is especially vital given the extraordinarily high burden of malaria in Uganda and the excellent efficacy of ACT when used for uncomplicated malaria in the country [16-21].

Not only was the decision to use ACT frequently appropriate, but nearly all patients prescribed ACT were prescribed AL, the recommended first-line therapy for uncomplicated malaria in Uganda. Artesunate plus amodiaquine, the alternate first-line therapy for uncomplicated malaria, was not prescribed during the study period and is not procured by the Uganda Ministry of Health. AN and DP, which comprised all but one of the remaining forms of ACT prescribed, were used at isolated sites during select months as small quantities of these medications had been made available by international donors during the study period. Further encouraging was the fact that stock-outs were rare as pharmacy-dispensing records indicated that 91.5% of prescribed anti-malarials were dispensed by the pharmacy in 2011 and 98.7% were dispensed in 2012. The rise in the proportion of anti-malarials dispensed from 2011 to 2012 is likely in large part due to changes in how medications were delivered to the health centers. Beginning in mid-2011 the National Medical Stores began directly delivering medications to the level IV health centers (bypassing the district health team) as part of a comprehensive effort to streamline the supply chain. This system has reduced the frequency of stock-outs being reported by the health facilities [13]. Improved data collection during this time period may also explain some of the rise in the proportion of anti-malarials dispensed as pharmacy practices were first incorporated into the surveillance system in January of 2011 and the proportion of records missing this information declined from 4.3% in 2011 to 1.1% in 2012.

The translation of ACT policy to ACT prescribing practices has been difficult in many African countries where rates of appropriate ACT use has ranged from 22% to 76% [22-29]. One study in Kenya did show that AL was prescribed to 90% of patients with uncomplicated laboratory-confirmed malaria when data were limited to health facilities with both AL and diagnostic testing in stock [4]. Various barriers to ACT adoption have been cited, including low numbers of clinicians with advanced degrees and inadequate provider training which often focused on in-service lectures and dissemination of written guidelines without chart audits, feedback, and follow-up training. Stock outs have also been shown to be a barrier to appropriate anti-malarial prescription practices in sub-Saharan Africa [30] and the low rates of stock outs at the facilities presented in this study may have contributed to the high rates of ACT use. These results stand in contrast to early reviews of data from selected health facilities in Uganda, which reported difficulty in achieving high rates of compliance with the new ACT treatment policy in the setting of frequent stock outs. A study done one year after the rollout of AL showed that AL was prescribed in only 60% of outpatient fever cases when it was indicated per national guidelines [10]. A single health facility study did, however, report rates as high as 89% during this same time-frame [31]. Also in contrast to the results presented here, neither of these studies reported high rates of diagnostic testing prior to anti-malarial prescription. The low utilization of malaria diagnostic testing appears to be common in Uganda as a national survey done in 2011 revealed that only 32% of children under five years of age had blood drawn when taken to a health facility for fever [32]. While an investigation using 2010 data taken from the same sentinel sites reported here described high rates of diagnostic testing, AL use was only 69% in outpatient laboratory-confirmed cases [11]. The findings described in this report thus indicate substantial improvement in ACT prescription practices at these sites over the last two years.

Despite the high rate of appropriate ACT use in the study, there is still a minority of ACT candidates who are not prescribed ACT. The data presented here suggest some variables associated with a failure to prescribe ACT to an appropriate candidate, but why these associations exist and what can be done to correct them will warrant further investigation. Certain clinical sites, for example, had higher rates of failure to appropriately prescribe ACT, but the reason for this discrepancy is uncertain. One Ugandan study noted a difference in prescribing practices between nurses and clinical officers or physicians [31], which could be a contributing factor in this study’s sites as the composition of clinic staff is different at each site. Differences in dissemination of training and local anti-malarial prescribing culture at each clinic may also be playing a role. The finding that younger children were at higher risk of being prescribed non-ACT when they were ACT candidates is surprising and also merits further study. Given the excellent efficacy and side effect profile of these medications, coupled with the increased vulnerability of young children to complications of malaria, correcting this practice will be of great importance. Additionally, women of childbearing age who are not pregnant should be prescribed ACT for uncomplicated malaria, yet multivariable analysis reveals that this group is at increased risk of not receiving ACT. This finding suggests that providers may be avoiding ACT out of fear that the patient might be or will soon become pregnant, a practice that is not endorsed by the Ugandan malaria treatment guidelines. Improved diagnosis of early pregnancy and additional ACT training interventions will be needed to target this remaining deficiency as well. Finally, the finding that those who were prescribed antibiotics were more likely to be prescribed ACT is surprising. It is not clear why this association exists and further studies will be needed to better understand this practice.

Among the patients who were not clear candidates for ACT, the proportion of patients with severe malaria or referral for admission who were prescribed quinine was very high (93.4%). Administration of quinine to these patients would be consistent with Ugandan malaria treatment guidelines assuming these patients did indeed have severe malaria. There was one subgroup of patients, however, who received therapy outside of national guidelines at high rates – those who were below age or weight criteria for ACT. These patients received a prescription for ACT more often than they received a prescription for quinine. Making providers aware of the weight and age minimums requisite to ACT prescription will be imperative.

There are several limitations to this study. The sentinel surveillance sites in Uganda are unique clinical settings where laboratory confirmation of malaria is common, thus limiting the generalizability of the findings. Also limiting generalizability is the fact that many patients do not seek treatment at public clinics, choosing instead to rely on private clinics, private pharmacies, and traditional healers [32,33]. Additionally, the classification of candidacy for ACT is dependent on the clinical judgment and documentation of the clinicians as well as the decision to admit the patient. These are all imperfect markers for identifying severe malaria. Finally, the data collected indicate what anti-malarial is prescribed and dispensed by the pharmacy but not the dosing of the medication, the route of administration, or the patient’s adherence to the prescribed regimen. While a single study showed that AL is usually dosed correctly in Uganda [10], reports on the adherence of Ugandans to anti-malarials have varied substantially [34-36] and no conclusions on dosing, route of administration, or adherence can be drawn from this study.

## Conclusions

More than six years after Uganda rolled out ACT as the first-line therapy for uncomplicated malaria, prescription practices show very high rates of compliance with recommended ACT use across six outpatient sites with high rates of malaria diagnostic testing and an improved supply chain of anti-malarials. The unique health facility-based malaria surveillance system operating at these clinical sites—which has provided additional clinical training, feedback on case management, and sharing of data collected from the sites—is likely a crucial component of the high rates of appropriate ACT use. Such improvements in clinical training and feedback may serve as a model for improving compliance with guidelines for ACT use at other sites in sub-Saharan Africa.

## Abbreviations

ACT: Artemisinin-based combination therapy; AL: Artemether-lumefantrine; AN: Artemisinin-naphthoquine; CRF: Case record form; DP: Dihydroartemisinin-piperaquine; GFATM: Global Fund for AIDS, Tuberculosis and Malaria; IQR: Inter-quartile range; NMCP: National Malaria Control Programme; PMI: President’s Malaria Initiative; SP: Sulphadoxine-pyrimethamine; RDT: Rapid diagnostic test; UMSP: Uganda Malaria Surveillance Project; WHO: World Health Organization.

## Competing interests

The authors declare that they have no competing interests.

## Authors’ contributions

GD, DS, and AM conceived and designed the study. RK, AM, SK, and AS participated in data collection. GD and DS performed the data analysis with input from all authors. All authors participated in the writing of the manuscript. All authors read and approved the final manuscript.

## Supplementary Material

Additional file 1 **Case record form.** Standardized form used to collect individual patient-level data during the clinical encounter. Click here for file
