# Supplementary material for: Anti-malarial prescription practices among outpatients with laboratory-confirmed malaria in the setting of a health facility-based sentinel site surveillance system in Uganda
Source: Malar J. 2013 Jul 19;12:252. doi: 10.1186/1475-2875-12-252 (PMC3723425; doi:10.1186/1475-2875-12-252)
Supplement: Additional file 1 — Case record form. Standardized form used to collect individual patient-level data during the clinical encounter. [file 1475-2875-12-252-S1.pdf]

Patient Record Form   Clinic______________

Date |___||___|/_|___||___|/|___||___|	OPD Number |___||___||___||___|	Patient's Last Name	First Name	New attendance ▢Yes ▢ No
Parish	Village	Age: |___||___| Yrs |___||___| Mos	Sex ▢Male ▢Female	Weight |___||___|.|___|kg

History & Exam Findings (complete **ALL** questions)

Fever or history of fever? ▢ Yes ▢ No	Cough? ▢ Yes ▢ No	Cough ≥ 2 weeks? ▢ Yes ▢ No	Weight loss? ▢ Yes ▢ No	Drenching night sweats? ▢ Yes ▢ No

▢**BS for Malaria** ▢**Pos** ▢ **Neg**<br>**Parasite density: ________________(if positive)**<br>▢**RDT for Malaria** ▢**Pos** ▢ **Neg**<br>**Malaria Lab number |___||___||___||___|**	▢**HIV test** ▢**CTRR** ▢ **CTR**<br>**HIV Lab number |___||___||___||___|**	▢**TB exam:** **Date collected:** **Type** **Date reported:**<br>1st smear ▢**Pos** ▢**Neg** ____/____/____ ▢LM ▢FM ____/____/____<br>2nd smear ▢**Pos** ▢**Neg** ____/____/____ ▢LM ▢FM ____/____/____<br>**TB Lab number |___||___||___||___|**
▢ **Stool ordered** - Results:	▢ **Urinalysis ordered** – Results:	▢**Hb** |___||___|.|___|g/dl<br>▢**VDRL test** ▢**Pos** ▢ **Neg**<br>▢**Other** (test/result)

Diagnoses (Check all that apply)

**Reportable Diseases**	**Infectious Diseases**	**Non-infectious Diseases**	**Maternal and Perinatal Diseases**
▢ Acute flaccid paralysis	▢ AIDS/HIV	▢ Alcohol and drug abuse	▢ Abortions
▢ Cholera	▢ Cough or Cold (no pneumonia)	▢ Anaemia	▢ Haemorrhage during pregnancy
▢ Dysentery	▢ Diarrhea- Acute	▢ Animal and Snake bites	▢ High BP during pregnancy
▢ Guinea worm	▢ Diarrhea- Persistent	▢ Asthma	▢ Obstructed labour
▢ Hemorrhagic fever	▢ Intestinal worms	▢ Cardiovascular- High BP	▢ Perinatal conditions in newborns
▢ Measles	▢ Leprosy	▢ Cardiovascular- Other	
▢ Meningitis (Meningococcal)	▢ Malaria (not during pregnancy)	▢ Childhood mental disorder	**Miscellaneous Diseases**
▢ Plague	▢ Malaria (during pregnancy)	▢ Diabetes Mellitus	▢ Death in OPD (no diagnosis)
▢ Rabies	▢ Meningitis (Non meningococcal)	▢ Epilepsy	▢ ENT Conditions
▢ Tetanus (0-28 days age)	▢ Onchocerciasis	▢ GI disorders (non infectious)	▢ Eye Conditions
▢ Yellow Fever	▢ Pelvic Inflammatory Disease	▢ Injuries—Road Traffic Accidents	▢ Skin Conditions
	▢ Pneumonia	▢ Injuries—Trauma of other Origin	▢ Oral Diseases and conditions
**Tuberculosis**	▢ Schistosomiasis	▢ Malnutrition- low weight for age	▢
▢ New TB case – No prior TB treatment	▢ Sleeping Sickness	▢ Malnutrition- severe	
▢ New TB case – Previous TB treatment	▢ STI	▢ Mental Illness- Anxiety	**Other Cough Diagnoses**
▢ Known TB Case – Med Refill	▢ Tetanus (over 28 days age)	▢ Mental Illness- Depression	▢ Acute Bronchitis/LRTI (no pneumonia)
▢ Extra-pulmonary TB	▢ Typhoid Fever	▢ Mental Illness- Mania	▢ Allergic Rhinitis
	▢ Urinary Tract Infections (UTI)	▢ Mental Illness- Schizophrenia	▢ COPD
**Other Diagnosis**		▢ Mental Illness- Other	▢ Heartburn

Treatment (Check all that apply). For antimalarial drugs, Tick DA if Drug is Available and given or Tick OS if Drug is Out of Stock or Tick ANG if Drug is Available but not Given

**Drug**	**Dose**	**DA**	**OS**	**ANG**	**Drug**	**Dose**
**Antimalarial**					**Other Drugs**	
▢		▢	▢	▢	▢ Aspirin	
▢ Quinine		▢	▢	▢	▢ Cough linctus	
▢ Chloroquine		▢	▢	▢	▢ Diazepam	
▢ Amodiaquine		▢	▢	▢	▢ Dexamethasone	
▢ SP		▢	▢	▢	▢ Diclofenac	
▢ Artesunate		▢	▢	▢	▢ Folic Acid	
▢ DP		▢	▢	▢	▢ Gentian violet	
▢		▢	▢	▢	▢ Hydrocortisone	
**Antimicrobials**					▢ Ibuprofen	
▢ Albendazole					▢ Magnesium	
▢ Amoxilicilin					▢ Multivitamin	
▢ Chloramphenicol					▢ Nystatin	
▢ Ciprofloxacin					▢ Paracetamol	
▢ Cloxacillin					▢ Phenytoin	
▢ Cotrimoxazole					▢ chlorampenicol	
▢ Doxycycline					▢ Salbutamol	
▢ Erythromycin					▢ Vit. B group	
▢ Gentamicin					▢	
▢ Mebendazole					Other	
▢ Metronidazole					Other	
▢ PPF					Other	
▢ Tetracycline					Other	
▢ X-pen					Other	

**Referrals and additional notes**		**TB Drug Regimen** (Check if prescribed)							
▢ Admitted to ward	Notes	**Initiation:**	**DA**	**OS**	**ANG**	**Continuation:**	**DA**	**OS**	**ANG**
▢ Referred to HIV care		▢ RHZE	▢	▢	▢	▢ HE	▢	▢	▢
▢ **Referred for TB care**		▢ RHZES	▢	▢	▢	▢ RHE	▢	▢	▢
▢ Referred for other services		▢ RHZ	▢	▢	▢	▢ RH	▢	▢	▢

Full Name   Signature
